# Association of the gut microbiome and different phenotypes of COPD and asthma: a bidirectional Mendelian randomization study

**DOI:** 10.1128/spectrum.01760-24

**Published:** 2024-10-07

**Authors:** Zihan Wang, Jingge Qu, Chun Chang, Yongchang Sun

**Affiliations:** 1 Department of Respiratory and Critical Care Medicine, Peking University Third Hospital, Research Center for Chronic Airway Diseases, Peking University Health Science Center, Beijing, China; Children's National Hospital, Washington, DC, USA

**Keywords:** Mendelian randomization, gut microbiota, early-onset COPD, later-onset COPD, allergic asthma, non-allergic asthma, causality

## Abstract

Mounting evidence has revealed the association between gut microbiota and both chronic obstructive pulmonary disease (COPD) and asthma; however, the causal association between gut microbiota and specific disease phenotypes remains to be determined. This study employed bidirectional two-sample Mendelian randomization (MR) analyses to investigate the potential causal relationship between gut microbiota and these conditions. The research utilized genome-wide association study (GWAS) data from the MiBioGen consortium for gut microbiota and the integrative epidemiology unit (IEU) Open GWAS for these conditions. Four MR analysis methods were employed: the inverse variance weighted (IVW) test, MR-Egger, weighted median, and weighted mode methods. The IVW method results are considered the primary findings. Sensitivity analyses, including heterogeneity tests, horizontal pleiotropy analysis, and leave-one-out analysis, were used to enhance robustness. Our MR study identified eight gut microbiota taxa potentially associated with the risk of different types of COPD and asthma. These include two taxa for early-onset COPD: *Streptococcaceae* [odds ratio (OR) = 1.315, 95% confidence interval (CI) = 1.071–1.616, *P* = 0.009] and *Holdemanella* (OR = 1.199, 95% CI = 1.063–1.352, *P* = 0.003); three for later-onset COPD: *Acidaminococcaceae* (OR = 1.312, 95% CI = 1.098–1.567, *P* = 0.003), *Holdemania* (OR = 1.165, 95% CI = 1.039–1.305, *P* = 0.009), and *Marvinbryantia* (OR = 0.814, 95% CI = 0.697–0.951, *P* = 0.009); one for allergic asthma: *Butyricimonas* (OR = 0.794, 95% CI = 0.693–0.908, *P* = 0.001); and two for non-allergic asthma: *Clostridia* (OR = 1.255, 95% CI = 1.043–1.511, *P* = 0.016) and *Clostridiales* (OR = 1.256, 95% CI = 1.048–1.506, *P* = 0.014).

**IMPORTANCE:**

Individuals with diverse phenotypes of chronic obstructive pulmonary disease (COPD) and asthma exhibit different responses to the conventional “one treatment fits all” approach. Recent research has revealed the significant role of the gut-lung axis in both COPD and asthma. However, the specific impact of gut microbiota on different subtypes of these conditions remains poorly understood. Our study has identified eight gut microbiota that may be associated with the risk of different types of COPD and asthma. These findings provide evidence suggesting a potential causal relationship between gut microbiota and various phenotypes of COPD and asthma. This offers a new perspective on the origins of different disease phenotypes and points toward future exploration of phenotype-specific and personalized therapies.

## INTRODUCTION

Chronic obstructive pulmonary disease (COPD) and asthma are prevalent chronic non-communicable airway disorders that affect over half a billion individuals globally while imposing significant societal burdens (1, 2). COPD is a heterogeneous disease with high morbidity and mortality, characterized by progressive inflammation and irreversible airflow restriction in both large and small airways (3), which can be classified into early-onset COPD, with an age of onset typically less than 50 years, and later-onset COPD, which primarily affects those aged over 60 years (4). Asthma is an intractable global health issue affecting all ages, characterized by wheezing, shortness of breath, chest tightness, cough, etc. (5). Unlike COPD, asthma is characterized by reversible airflow restriction and variable respiratory symptoms due to recurrent airway inflammation and remodeling, which can be further divided into allergic (type 2) and non-allergic (non-type 2) asthma, depending on immunological and molecular markers (6–9). Although it has been recognized that the etiology of COPD and asthma results from environmental and genetic interactions, the etiology of these two diseases, particularly for concerned phenotypes, has not yet been fully clarified (10, 11). Meanwhile, clinical features and therapeutic regimens vary among patients with diverse phenotypes of these two specific diseases (4, 12, 13). In addition, despite using an aggressive step-up treatment regimen for asthma and COPD patients, many continue to experience uncontrolled symptoms (14, 15). Collectively, the exploration of the specific etiology of phenotypes of COPD and asthma can provide novel prevention and treatment insights for individualized and phenotype-specific therapy with the advance of precision medicine.

A variety of microorganisms colonizes the human digestive tract called the gut microbiota, which can directly interact with host interstitial cells and perform several functions, such as vitamin production, absorption of essential ions, resistance to invading pathogens, and even the establishment of immune responses, etc. (16–18). Although the traditional notion is that gut colonizing microbiota was conserved in the host body, mounting research recently revealed imbalances of host microorganisms between the healthy and patients with certain respiratory diseases. For instance, a large prospective cohort study lasting up to 15 years found the diversity of gut microbiota between people who suffered from asthma and COPD and healthy people (19). However, the causality between these microbial differences and the disease remains elusive. Therefore, it is imperative further to elucidate the interaction mechanism between intestinal flora and lung and improve the gut-lung axis system, providing a new direction for understanding diverse phenotypes of asthma and COPD in terms of etiology and pathogenesis.

Mendelian randomization (MR) is an emerging research method to explore the relationship between exposure and outcome, as high-quality randomized controlled studies consist of large samples with significant shortcomings in time and cost consumption (20). Alternatively, genome-wide association studies (GWASs) are used to identify sequence variations, known as single nucleotide polymorphisms (SNPs), across the entire human genome to screen out disease-associated SNPs, which are further to be used as instrumental variables (IVs) in MR analysis while eliminating the influence of other confounding factors and avoiding reverse causation (21, 22). Although previous MR studies have investigated the causal relationship between gut microbiota and chronic airway diseases, for instance, asthma and COPD by Shi et al. (23) and Wei et al. (24), studies on the specific phenotypes of both disorders are still scarce.

In this study, we innovatively investigated the potential causal relationship between intestinal flora and diverse phenotypes of COPD and asthma, providing a novel idea for the etiology of different phenotypes of these diseases and opening further scenarios for subsequent phenotype-specific and individualized therapy.

## RESULTS

### Selection of instrumental variables

Through screening intestinal flora data, with *P* < 1 × 10^−05^ as the threshold and removing linkage disequilibrium (LD) and *F* statistics lower than 10, a total of 2,513 eligible SNPs were screened from 196 intestinal flora taxa as IVs finally, including 9 phyla (123 SNPs), 16 class (221 SNPs), 20 order (276 SNPs), 31 families (428 SNPs), and 117 genera (1,465 SNPs) (Table S1). Additionally, SNPs affecting the outcome, such as lung function indicators or smoking status, were eliminated as potential confounding factors by consulting the Phenoscanner V2 database, and detailed information for diverse concerned outcomes is presented in Tables S2 to S5.

### The causal relationship between gut microbiota and early-onset COPD

**Fig 1:**
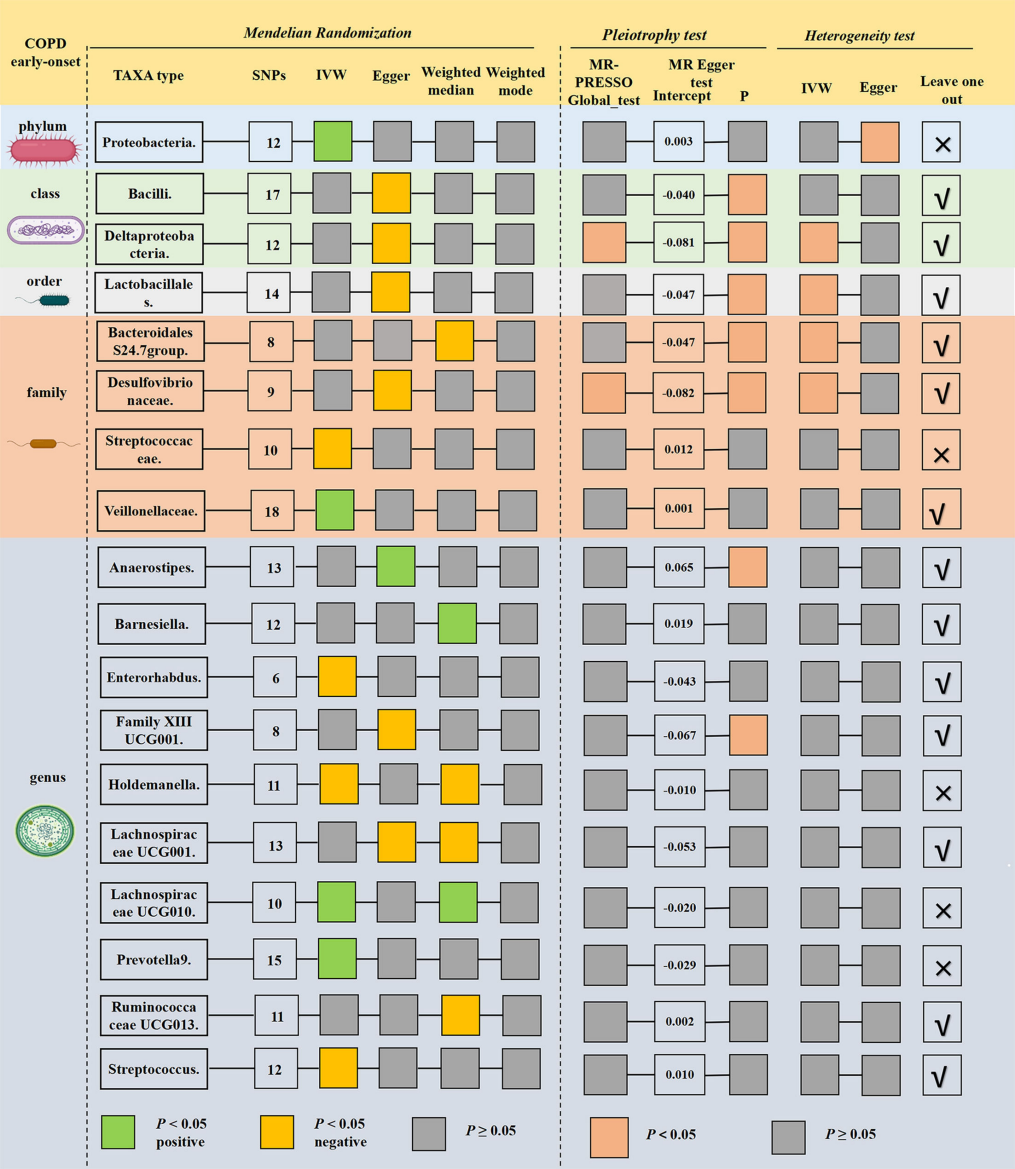
Preliminary MR results and sensitivity analysis of the significant relationship between gut microbiota and early-onset COPD. In the image, the green box on the left indicates a positive relationship between the exposure being studied and the outcome with statistically significant results. On the other hand, the orange box represents a negative relationship between the two. The pink box on the right shows the statistical significance of the sensitivity analysis results. The number on the far right indicates whether any SNPs were removed due to the leave-one-out analysis. An “X” means that no SNP was removed, while the “√” indicates that there were SNPs that could have biased the results.

Table S6 presented all the results of the MR study between all gut microbiota taxa and early-onset COPD. Figure 1 shows eight gut microbiota taxa that may be causally associated with early-onset COPD analyzed by the TSMR based on the inverse variance weighted (IVW) method, where one was phylum part, two were family part, and five were genus part. Among them, the genetically predicted phylum *Proteobacteria* [odds ratio (OR) = 0.754; 95% confidence interval (CI), 0.591–0.961, *P* = 0.023], family *Veillonellaceae* (OR = 0.869; 95% CI, 0.762–0.992, *P* = 0.037), genus *Lachnospiraceae UCG010* (OR = 0.788; 95% CI, 0.652–0.951, *P* = 0.013), and genus *Prevotella9* (OR = 0.858; 95% CI, 0.761–0.968, *P* = 0.013) could exert a protective factor for early-onset COPD. Besides, the genetically predicted family *Streptococcaceae* (OR = 1.315; 95% CI, 1.071–1.616, *P* = 0.009), genus *Enterorhabdus* (OR = 1.267; 95% CI, 1.029–1.559, *P* = 0.026), genus *Holdemanella* (OR = 1.199; 95% CI, 1.063–1.351, *P* = 0.003), and genus *Streptococcus* (OR = 1.246; 95% CI, 1.017–1.527, *P* = 0.034) were associated with a high risk of early-onset COPD.

Sensitivity analyses of eight gut microbiota taxa that may have a causal relationship with early-onset COPD are presented in Fig. 1 and Table S7. Cochran’s Q and Rucker’s *Q* statistics revealed the exhibition of heterogeneity between *Proteobacteria* and early-onset COPD studies. The leave-one-out analysis demonstrated that specific SNPs could lead to some bias for three gut microbiota taxa, *Veillonellaceae*, *Enterorhabdus*, and *Streptococcus* in genetic prediction (Fig. S1).

Above all, following MR Analysis and multi-aspect sensitivity analysis, a potential causal relationship was observed between two types of intestinal flora namely the family *Streptococcaceae* and genus *Holdemanella* with early-onset COPD based on the IVW method (Table 1).

**TABLE 1:** MR results of the significant relationship between gut microbiota and early-onset COPD^*a*^

Classification	Exposure	Method	nSNP	OR	95% CI	*P*
Family	*Streptococcaceae*	**IVW**	10	**1.315**	**1.071–1.616**	**0.009**
		MR-Egger		1.278	0.548–2.981	0.586
		Weighted median		1.205	0.900–1.614	0.210
		Weighted mode		1.135	0.714–1.804	0.604
Genus	*Holdemanella*	**IVW**	11	**1.199**	**1.063–1.352**	**0.003**
		MR-Egger		1.300	0.922–1.834	0.169
		**Weighted median**		**1.199**	**1.025–1.402**	**0.023**
		Weighted mode		1.226	0.958–1.569	0.137

^*a*^ Results with *P* < 0.05 are highlighted in bold.

### The causal relationship between gut microbiota and later-onset COPD

The results of the MR study within the causality are demonstrated in Table S8. The MR analysis indicated that four gut microbiota taxa could have a causal relationship with later-onset COPD mainly based on the IVW method. The genetically predicted family *FamilyXI* (OR = 0.915; 95% CI, 0.840–0.997, *P* = 0.042) and genus *Marvinbryantia* (OR = 0.814; 95% CI, 0.697–0.951, *P* = 0.009) could be a protective factor for the development of COPD. In contrast, the genetically predicted family *Acidaminococcaceae* (OR = 1.312; 95% CI, 1.098–1.567, *P* = 0.003) and genus *Holdemania* (OR = 1.164; 95% CI, 1.039–1.305, *P* = 0.009) could exert a negative effect on later-onset COPD.

**Fig 2:**
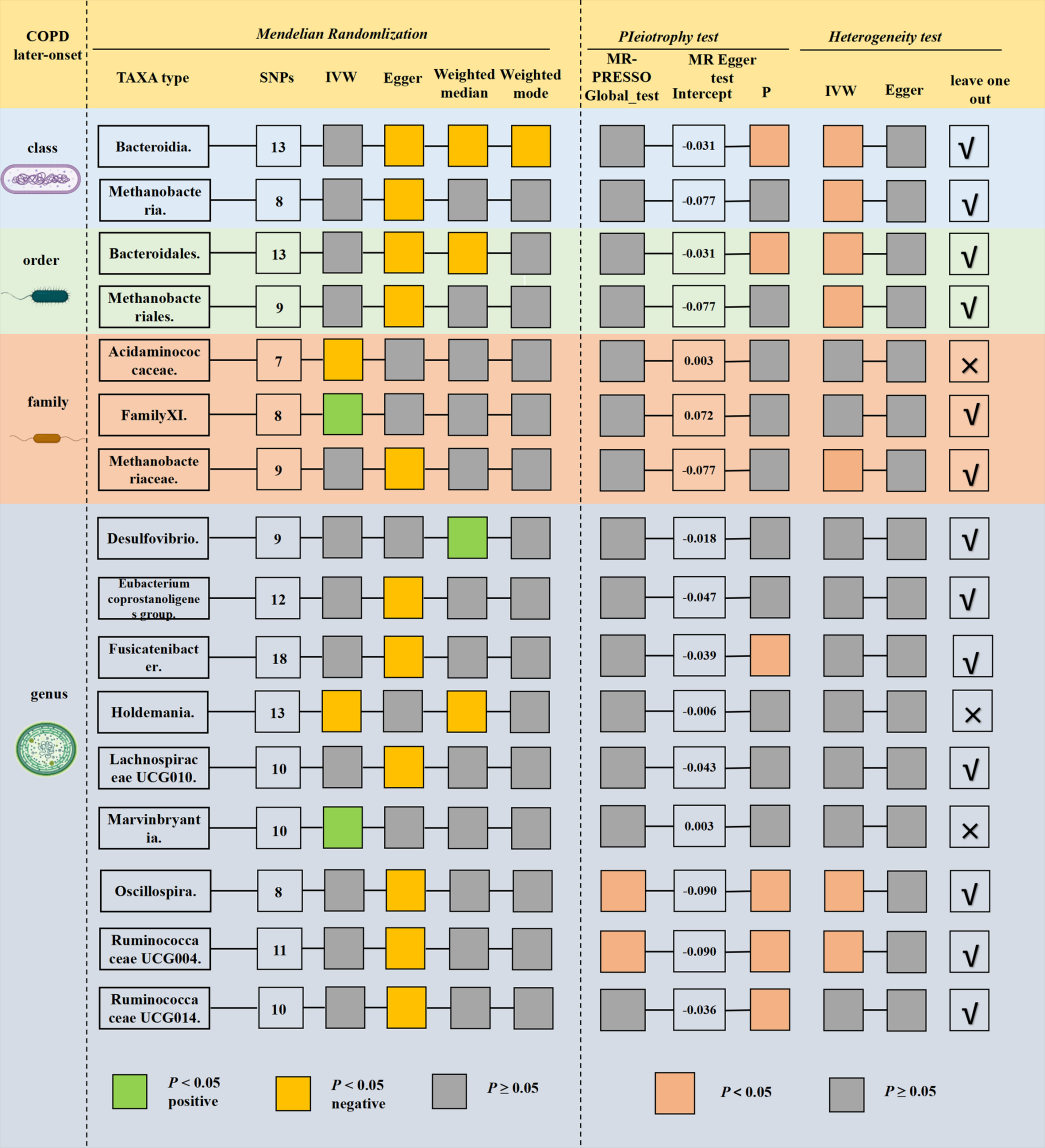
MR results and sensitivity analysis of significant relationship between gut microbiota and later-onset COPD.

Figure 2 and Table S9 show the sensitivity analysis results of the four gut microbiota taxa. No significant heterogeneity and horizontal pleiotropy were observed in the outcome of the above four gut microbiota taxa. The leave-one-out analysis revealed that specific SNPs could lead to some bias for *FamilyXI* gut microbiota taxa (Fig. S2).

Ultimately, our comprehensive analysis revealed that three taxa, namely *Acidaminococcaceae*, *Holdemania and Marvinbryantia* could be associated with the development of later-onset COPD without exhibiting any horizontal pleiotropy or heterogeneity (Table 2).

**TABLE 2:** MR results of the significant relationship between gut microbiota and later-onset COPD^*a*^

Classification	Exposure	Method	nSNP	OR	95% CI	*P*
Family	*Acidaminococcaceae*	**IVW**	7	**1.312**	**1.098–1.567**	**0.003**
		MR-Egger		1.276	0.706–2.304	0.457
		Weighted median		1.177	0.937–1.479	0.161
		Weighted mode		1.174	0.879–1.567	0.320
Genus	*Holdemania*	**IVW**	13	**1.165**	**1.039–1.305**	**0.009**
		MR-Egger		1.236	0.875–1.746	0.254
		**Weighted median**		**1.188**	**1.015–1.391**	**0.032**
		Weighted mode		1.255	0.960–1.641	0.122
	*Marvinbryantia*	**IVW**	10	**0.814**	**0.697–0.951**	**0.009**
		MR-Egger		0.791	0.431–1.453	0.472
		Weighted median		0.833	0.673–1.032	0.095
		Weighted mode		0.847	0.648–1.106	0.254

^*a*^ Results with *P* < 0.05 are highlighted in bold.

### The causal relationship between gut microbiota and allergic asthma

**Fig 3:**
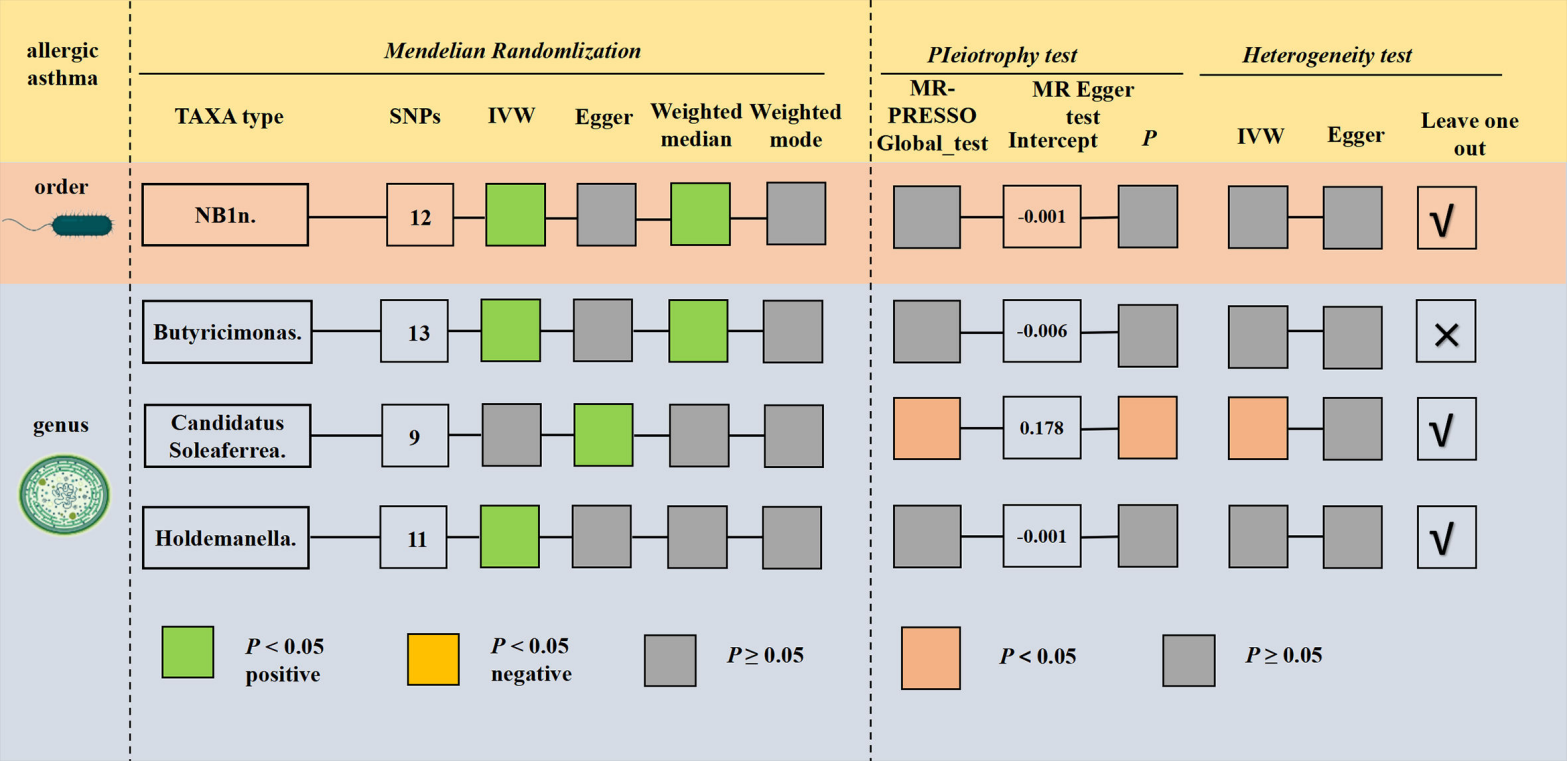
Preliminary MR results and sensitivity analysis of significant relationship between gut microbiota and allergic asthma.

For allergic asthma, only three taxa presented possible correlation (Fig. 3; Table S10). The genetically predicted order *NB1n* (OR = 0.894; 95% CI, 0.817–0.977, *P* = 0.014), genus *Butyricimonas* (OR = 0.794; 95% CI, 0.693–0.908, *P* = 0.001), and genus *Holdemanella* (OR = 0.890; 95% CI, 0.795–0.997, *P* = 0.044) could be lower risk factors for early-onset COPD based on the IVW test.

Sensitivity analysis presented non-negligible horizontal pleiotropy or heterogeneity for *Candidatus Soleaferrea* (Fig. 3; Table S11). Meanwhile, the leave-one-out analysis also indicated that some SNPs could lead to deviation in the result for three taxa except *Butyricimonas* (Fig. S3).

Collectively, after excluding the effects of heterogeneity and horizontal pleiotropy, our finding revealed that those genetically predicated to *Butyricimonas* are likely to have a reduced risk of allergic asthma (Table 3).

**TABLE 3:** MR results of the significant relationship between gut microbiota and allergic asthma^*a*^

Classification	Exposure	Method	nSNP	OR	95% CI	*P*
Genus	*Butyricimonas*	**IVW**	13	**0.794**	**0.693–0.908**	**0.001**
		MR-Egger		0.850	0.527–1.373	0.521
		**Weighted median**		**0.804**	**0.662–0.975**	**0.026**
		Weighted mode		0.830	0.612–1.125	0.252

^*a*^ Results with *P* < 0.05 are highlighted in bold.

### The causal relationship between gut microbiota and non-allergic asthma

**Fig 4:**
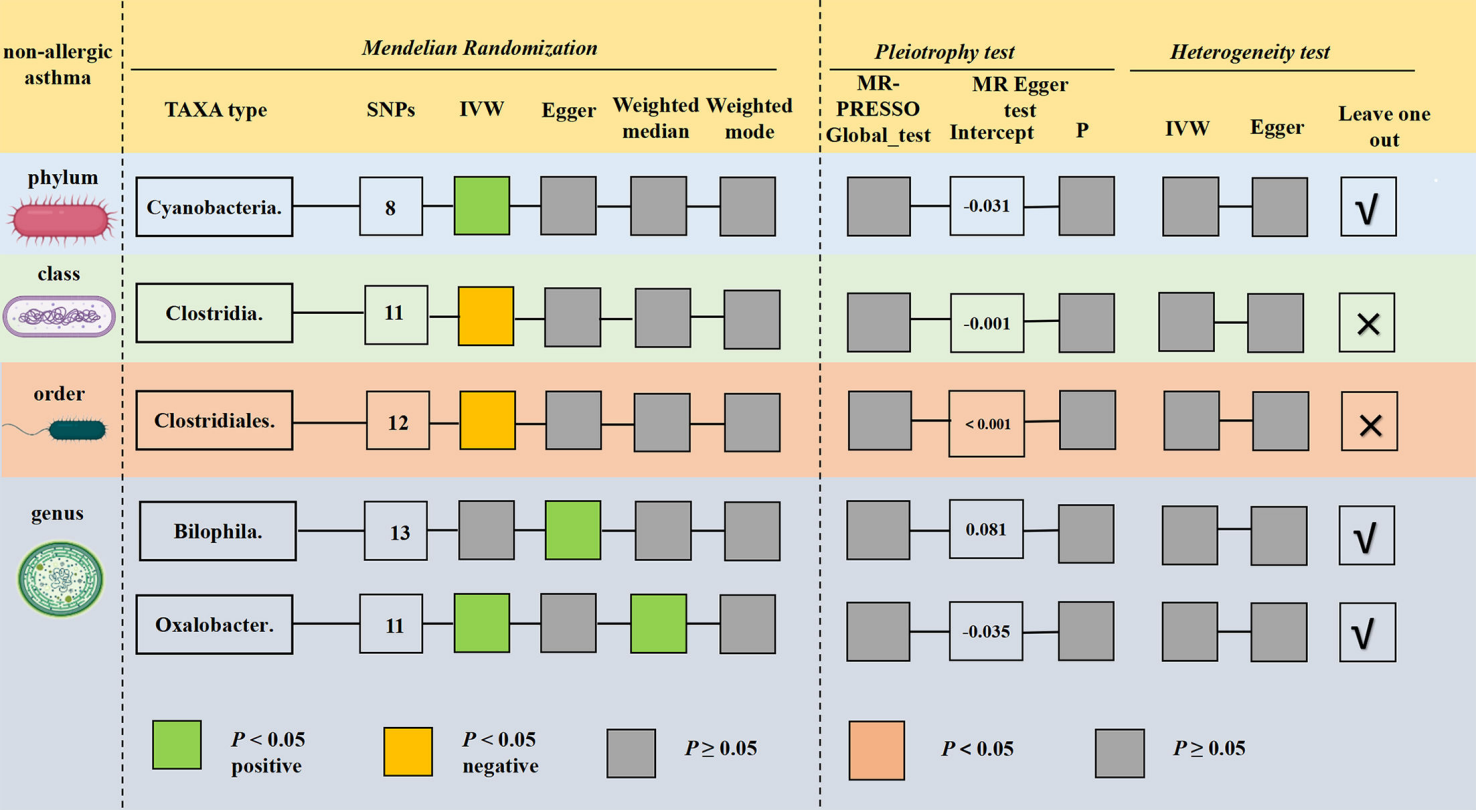
Preliminary MR results and sensitivity analysis of the significant relationship between gut microbiota and non-allergic asthma.

Next, four gut microbiota taxa that could be causally related to non-allergic asthma were discovered by MR analysis primarily based on the IVW method (Fig. 4; Table S12). The results indicated that genetically predicated phylum *Cyanobacteria* (OR = 0.848; 95% CI, 0.731–0.983, *P* = 0.003) and genus *Oxalobacter* (OR = 0.872; 95% CI, 0.790–0.962, *P* = 0.007) could reduce the risk of non-allergic asthma. In contrast, a higher level of class *Clostridia* (OR = 1.255; 95% CI, 1.042–1.511, *P* = 0.016) and order *Clostridiales* (OR = 1.256; 95% CI, 1.048–1.506, *P* = 0.014) could elevate the risk of developing non-allergic asthma.

Sensitivity analysis revealed that specific SNPs could lead to some bias for two gut microbiota taxa: *Cyanobacteria* and *Oxalobacter* (Table S13; Fig. S4). As a result, it can be concluded that genetically predicated *Clostridia* and *Clostridiales* could elevate the risk of non-allergic asthma (Table 4).

**TABLE 4:** MR results of the significant relationship between gut microbiota and non-allergic asthma^*a*^

Classification	Exposure	Method	nSNP	OR	95% CI	*P*
Class	*Clostridia*	**IVW**	11	**1.255**	**1.043–1.511**	**0.016**
		MR-Egger		1.276	0.783–2.081	0.354
		Weighted median		1.279	0.995–1.644	0.054
		Weighted mode		1.510	1.038–2.196	0.057
Order	*Clostridiales*	**IVW**	12	**1.256**	**1.048–1.506**	**0.014**
		MR-Egger		1.251	0.774–2.024	0.382
		Weighted median		1.210	0.943–1.554	0.134
		Weighted mode		1.476	0.972–2.242	0.095

^*a*^ Results with *P* < 0.05 are highlighted in bold.

### Reverse MR analysis

Through reverse MR, we identified that individuals with early COPD can impact four types of intestinal flora, while those with advanced COPD can affect 10 types. Individuals with allergic asthma and non-allergic asthma were found to influence 14 and 11 types of intestinal flora, respectively. After testing for horizontal pleiotropy and heterogeneity, only four intestinal floras in allergic asthma were excluded. No studies were removed based on leave-one-out analysis (Tables S14 to S21; Fig. S5 to S8).

## DISCUSSION

Previous studies have widely employed MR analysis to establish the causal relationship between specific gut microbiota and COPD and asthma (23, 24). However, asthma and COPD are two common heterogeneous diseases, and accurately categorizing them is essential in determining the most effective treatment and predicting long-term outcomes. Therefore, we employed MR to investigate the association between gut microbiota and distinct phenotypes of COPD and asthma. In this study, we have indicated that eight gut microbiota taxa could be associated with the occurrence and the development of various phenotypes of COPD and asthma using large-scale GWAS statistics. Additionally, reverse MR analysis also indicated that genetic liability to different types of COPD and asthma also could affect the proportion of intestinal flora, resulting in an imbalance of gut flora. The findings of this study suggest that the relevant intestinal flora not only could serve as a critical indicator for the diagnosis and early recognition of the latent risk for diseases mentioned above, but also represent a promising and novel target for their prevention and treatment in the future.

The concept of the gut-lung axis, which elucidates the interconnected mucosal immune system between the gastrointestinal tract and lung, implicates a pivotal role for the gut microbiome in regulating inflammation in both acute and chronic respiratory diseases, including COPD and asthma (25, 26). Several biological and population studies have found that gut microbiota imbalance is associated with COPD and asthma progression. For instance, a recent experiment transplanted the fecal microbiota from patients with COPD into mice, and the recipient mice exhibited elevated lung inflammation (27). Additionally, colonization by *Clostridium difficile* at age 1 month was associated with wheezing and eczema throughout early childhood and with asthma at age 6–7 years, which further proved the role of gut microbiota dysbiosis in lung inflammation (28). At the same time, respiratory diseases themselves can also lead to microbial imbalance, resulting in gastrointestinal disorders (29, 30). Nevertheless, the exact mechanism of the connection between the gut and the lung has not been fully explored. It is hypothesized that the activated immune cells located in the gut can travel through the blood and lymphatic system to the lungs to exert a local immune response (31). Besides, short-chain fatty acids, extensively investigated bacterial metabolites composed mainly of propionic acid, butyric acid, and acetic acid produced by fermentation of undigested food from intestinal flora (32), have been shown in recent years to play a role in regulating airway inflammation and lung homeostasis through the gut-lung axis (33). They exert their effects on the lung mainly through two pathways: firstly, via blood circulation to bone marrow, where they modulate immune cell distribution and proportion (34); secondly, directly affecting lung tissue cells and immune cells as pro-inflammatory or anti-inflammatory mediators (35). Meanwhile, respiratory diseases themselves can induce the production of various cytokines, such as interleukin (IL)-6, IL-1β, and tumor necrosis factor (TNF) α, that exert effects on the intestinal tract (36–38), thereby disrupting the balance of gut microbiota and potentially influencing the subsequent development of gastrointestinal disorders (39).

In the case of early-onset COPD, two specific types of intestinal flora, namely family *Streptococcaceae* and genus *Holdemanella*, exhibit significant associations and are identified as potential risk factors for early-onset COPD. The family *Streptococcaceae* consists of three genera: *Streptococcus*, *Lactococcus*, and *Lactovum* (40). Previous observational research showed that, compared to the fecal microbiota in COPD patients from healthy controls, the genus *Streptococcus* was significantly elevated in COPD patients and negatively correlated with lung function (41). Interestingly, previous studies showed that the genus *Streptococcus* was more prevalent in childhood asthma and young overweight/obese individuals (42, 43), implying its potential contribution to the development of early-onset COPD. Besides, recent research has suggested a potential connection between the abundance of *Streptococcus* in the gut and the levels of high-sensitivity C-reactive protein, lymphocytes, and neutrophils. This correlation may indicate that *Streptococcus* could potentially be the risk factor for early-onset COPD by triggering an inflammatory response in the human body. Additionally, it is important to note that no previous studies have investigated the potential link between the genus *Holdemanella* and lung diseases such as COPD. However, previous research indicated that there is a negative relationship between the abundance of *Holdemanella* and propionate levels in patients with diabetes and cognitive impairment (44). Propionate has been shown to have a role in repairing damaged airway epithelium (45). As a result, it is conceivable that *Holdemanella* may play a role in the onset of early-stage COPD by influencing propionate. However, the precise mechanism by which it contributes to the occurrence of early-stage COPD is unclear, and more experiments are necessary to validate this conclusion.

In terms of later-onset COPD, similar to a previous MR study conducted by Wei et al. (24), the presence of the genus *Marvinbryantia* has been identified as a beneficial factor that can attenuate the development of COPD. Our analysis provides more accurate evidence highlighting its significance specifically in later-onset COPD. As a member of butyrate-producing species, the genus *Marvinbryantia* is capable of producing butyric acid, which has been previously demonstrated to mitigate inflammatory responses effectively (46, 47). In addition, our MR analysis revealed that the genus *Holdemania* and family *Acidaminococcaceae* may be the risk factors for late-onset COPD. And several health concerns, such as gout and Parkinson’s disease, have been proven to be associated with the genus *Holdemania* (48, 49). Furthermore, previous research has demonstrated that the genus *Holdemania* possesses the ability to degrade mucin, a vital constituent of the intestinal mucosa (50). This excessive degradation of mucin could lead to damage in the intestinal barrier and trigger a systemic inflammatory response, potentially contributing to its association with the occurrence of later-onset COPD. Although previous MR studies have suggested a potential elevation in disease susceptibility associated with the *Acidaminococcaceae* family (51, 52), the precise underlying mechanism remains unclear, especially for late-onset COPD.

Our MR analysis revealed that genetically predicated three distinct types of gut microbiota that could influence the occurrence and development of allergic and non-allergic asthma. Firstly, by comparing the intestinal flora of mice in the treated and non-treated groups of allergic asthma, Zhou et al. (53) demonstrated an increased abundance of the *Butyricimonas* genus in the treated group compared to the non-treated group, suggesting its potential protective effect against allergic asthma, which is consistent with our MR analysis. Additionally, our findings indicated that individuals with a genetic predisposition to the *class Clostridiales* and its affiliated *Clostridiales* are at an elevated risk of developing asthma, particularly non-allergic asthma. These results provide further evidence supporting the significant role of *Clostridiales* in the pathogenesis of non-allergic asthma. Interestingly, the findings from a recent double-blind study revealed that the administration of probiotics to asthma patients resulted in improved asthma symptoms and a reduction in the abundance of *Clostridiales* within the gut microbiota, thereby suggesting a potential promotive role of *Clostridiales* in asthma.

However, there are still some limitations in this study. Firstly, the use of GWAS data on gut microbiota as a tool for IVs with lower thresholds (*P* < 1 × 10^−05^) may have impacted the accuracy of the results. Secondly, due to the exclusive availability of GWAS data for subtypes of COPD and asthma in the FinnGen database, we were unable to access GWAS data from alternative sources for further validation of our findings. Thirdly, since the majority of participants in the GWAS meta-analysis for gut microbiota data were of European descent, potential interference from population stratification should be noted. As such, the results of this study may not be fully generalizable to individuals of non-European descent. In the foreseeable future, it is imperative to undertake additional genome-wide studies focusing on non-European ancestry, which will contribute to a more comprehensive comprehension of disease etiology, prevention, and treatment modalities across diverse ethnic backgrounds. Fourthly, it is crucial to acknowledge that the causal links between certain gut microbiota and specific subtypes of COPD and asthma identified through our MR studies are yet to be substantiated by experimental studies. Most of the previous studies primarily rely on comparing variations in gut flora between individuals with specific illnesses and those who are healthy, which does not provide adequate evidence to establish a causal relationship.

## MATERIALS AND METHODS

### Study design

**Fig 5:**
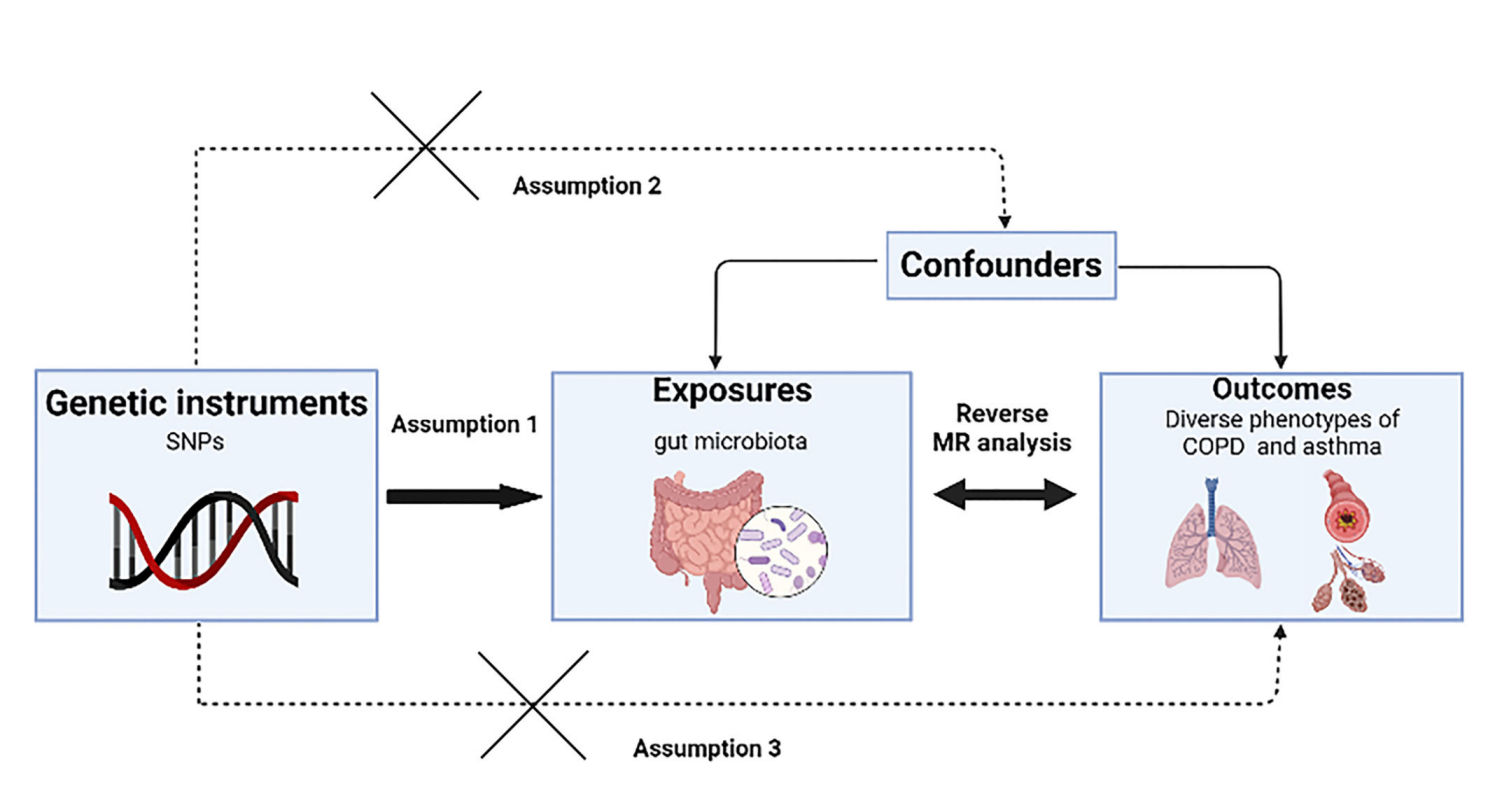
Overall design of the MR analysis.

Bidirectional two-sample Mendelian randomization (TSMR) was employed to explore the causal-effect relationship between gut microbiota and diverse phenotypes of COPD and asthma with SNPs as IVs. This study is reported following the STROBE-MR guidelines (Table S22). The overall design of this study is presented in Fig. 5.

### Data sources for exposure and outcome

The gut microbiota GWAS data were derived from 18,340 individuals of all ages in 11 regions analyzed by the MiBioGen consortium, the majority of whom had European ancestry (54). The study focused on variable regions V4, V3–V4, and V1–V2 of the 16S rRNA gene to profile microbial composition and conduct taxonomic classification using direct taxonomic binning. Microbiota quantitative trait loci mapping analysis was employed to pinpoint genetic loci associated with the abundance levels of bacterial taxa in the gut microbiota. The microbiota was divided into five levels, 211 taxas, namely 9 phyla, 16 classes, 20 orders, 35 families, and 131 genera, of which 15 unknown taxas were not included in this study (54). Additionally, covariates such as sex and age were adjusted for all participants. GWAS summary data for the different phenotypes of COPD (early-onset and later-onset COPD) and asthma (allergic and non-allergic asthma) were obtained from the latest ninth version of the FinnGen Research public data set (https://r9.finngen.fi/) released on 11 May 2023 (55). The participants involved were diagnosed based on the International Classification of Disease (ICD), tenth version ICD-10. The individuals' sex, age, the first 10 principal components, and genotyping batch also underwent a correction in the FinnGen data set. The detailed information on the GWAS is listed in Table 5. Other detailed demographic data available in the database are shown in Table S23.

**TABLE 5:** Characteristics of the GWAS used for analyses

Exposure	Source	Phenotype definition	No. cases	No. controls
Early-onset COPD	FinnGen-9	ICD-10 (J44) age < 65 years	7,079	359,794
Later-onset COPD	FinnGen-9	ICD-10 (J44) age ≥ 65 years	10,404	161,813
Allergic asthma	FinnGen-9	ICD-10 (J45.0)	9,631	210,122
Non-allergic asthma	FinnGen-9	ICD-10 (J45.1)	7,143	202,399
Gut microbiota	MiBioGen	NA	18,340	

### Genetic instrumental variables selection

The selection of eligible SNPs as IV is crucial for MR Analysis. Our analysis is based on previously established standards (20). The fundamental criteria for IVs to fulfill the MR assumptions in this research, as depicted in Fig. 5, are as follows: (i) the IVs must be associated with the exposure; (ii) the IVs must not be associated with any confounders of the exposure-outcome association; and (iii) the IVs should not influence the outcome, except possibly via its association with the exposure (20). In order to ensure the accuracy of the results of the causal relationship between gut microbiomes and different types of asthma and COPD, we selected *P* < 1 × 10^−05^ as the screening threshold for IVs commonly used in previous studies of gut microbiomes (56, 57). To fulfill assumption 1 of MR, we applied a stringent LD correlation coefficient threshold of *R*^2^ < 0.001 and a clumping window width of 10,000 kb. *R*^2^ for each instrument variant: *R*^2^ = 2 × EAF × (1 − EAF) × *β*^2^, where EAF is the effect allele frequency, and the *β* represents the effect size of the exposure instrument. Furthermore, we excluded SNPs that were associated with confounders or outcomes, such as smoking status, and lung function, according to the comprehensive retrieved results from the Phenoscanner V2 database and GWAS catalog to fulfill assumptions 2 and 3 (58). The general consensus is that when the *F* statistics exceed a threshold of 10, it indicates a low risk of weak IV bias. As a result, to mitigate the impact of weak IVs on the experimental outcomes, we excluded the SNPs with *F* < 10 computed using the formula *F* = *β*^2^exposure/SE^2^exposure (59), the SE represents the standard error of the exposure instrument.

### Reverse Mendelian randomization analyses

Reverse MR analyses were performed to investigate the potential impact of different types of COPD and asthma on gut microbiota. To enhance the accuracy of analysis results, we adjusted the IV threshold to 5 × 10^−07^, as SNPs obtained for non-allergic asthma at this level were insufficient for subsequent analysis (the threshold was set at 5 × 10^−06^). Other methods used in forward MR analysis remained unchanged.

### Statistical analysis

Consistent with previous MR studies (60–63), the IVW method was employed as the primary approach for conducting MR analysis. This meta-method synthesizes Wald estimates of each SNP, effectively treating each SNP as an effective natural experiment leading to more conservative and realistic parameter estimation, similar to two-stage least squares (64). Other complementary methods were also employed, such as MR-Egger regression, weighted median, and weighted mode. The MR-Egger regression can be used even when all SNPs are invalid. It estimates the causal effect through a weighted linear regression of gene-outcome coefficients on the gene-exposure coefficient (65). The weighted median approach yields consistent effect estimates when at least half of the weighted variance attributable to horizontal pleiotropy is valid (66). The weighted mode estimation assumes a plurality of genetic variants to be valid (67). However, these complementary analysis methods are vulnerable to the potential influence of invalid SNPs, which can introduce bias and inflate type I errors (66).

Additionally, a series of sensitivity analyses were conducted to ensure the results’ reliability. The horizontal pleiotropy tests were used to detect whether the selected IVs caused bias by directly influencing the outcome. Two methods were used to test the horizontal pleiotropy: the MR-PRESSO global test and the MR-Egger intercept test, *P* < 0.05 indicating the presence of horizontal pleiotropy (68). Besides, the degree of directional pleiotropy can be estimated by the intercept of the MR regression test (65). In addition, due to the heterogeneity of data sources, processing methods, etc., Cochran’s *Q* statistic (MR-IVW) and Rucker’s *Q* statistic (MR-Egger) were utilized to identify heterogeneity in this MR analysis, and *P* < 0.05 indicated that there existed heterogeneity between the studies (69, 70). Furthermore, leave-one-out sensitivity analyses were also employed to detect whether a single SNP can significantly influence the outcome.

The “TwosampleMR” package (version 0.5.7) of R software (version 4.2.2) was utilized to analyze the causal-effect relationship between the gut microbiota and diverse types of COPD and asthma using the IVs.

## SUPPLEMENTAL MATERIAL

Supplemental figures spectrum.01760-24-s0001.pdf The results of leave one out analysis.

Supplemental tables spectrum.01760-24-s0002.xlsx The data involved in MR Analysis and the results of sensitivity analysis.

ASM does not own the copyrights to Supplemental Material that may be linked to, or accessed through, an article. The authors have granted ASM a non-exclusive, world-wide license to publish the Supplemental Material files. Please contact the corresponding author directly for reuse.
