# Supplementary material for: Association of the gut microbiome and different phenotypes of COPD and asthma: a bidirectional Mendelian randomization study
Source: Microbiol Spectr. 2024 Oct 7;12(11):e01760-24. doi: 10.1128/spectrum.01760-24 (PMC11537028; doi:10.1128/spectrum.01760-24)
Supplement: Supplemental figures — The results of leave one out analysis. [file spectrum.01760-24-s0001.pdf]

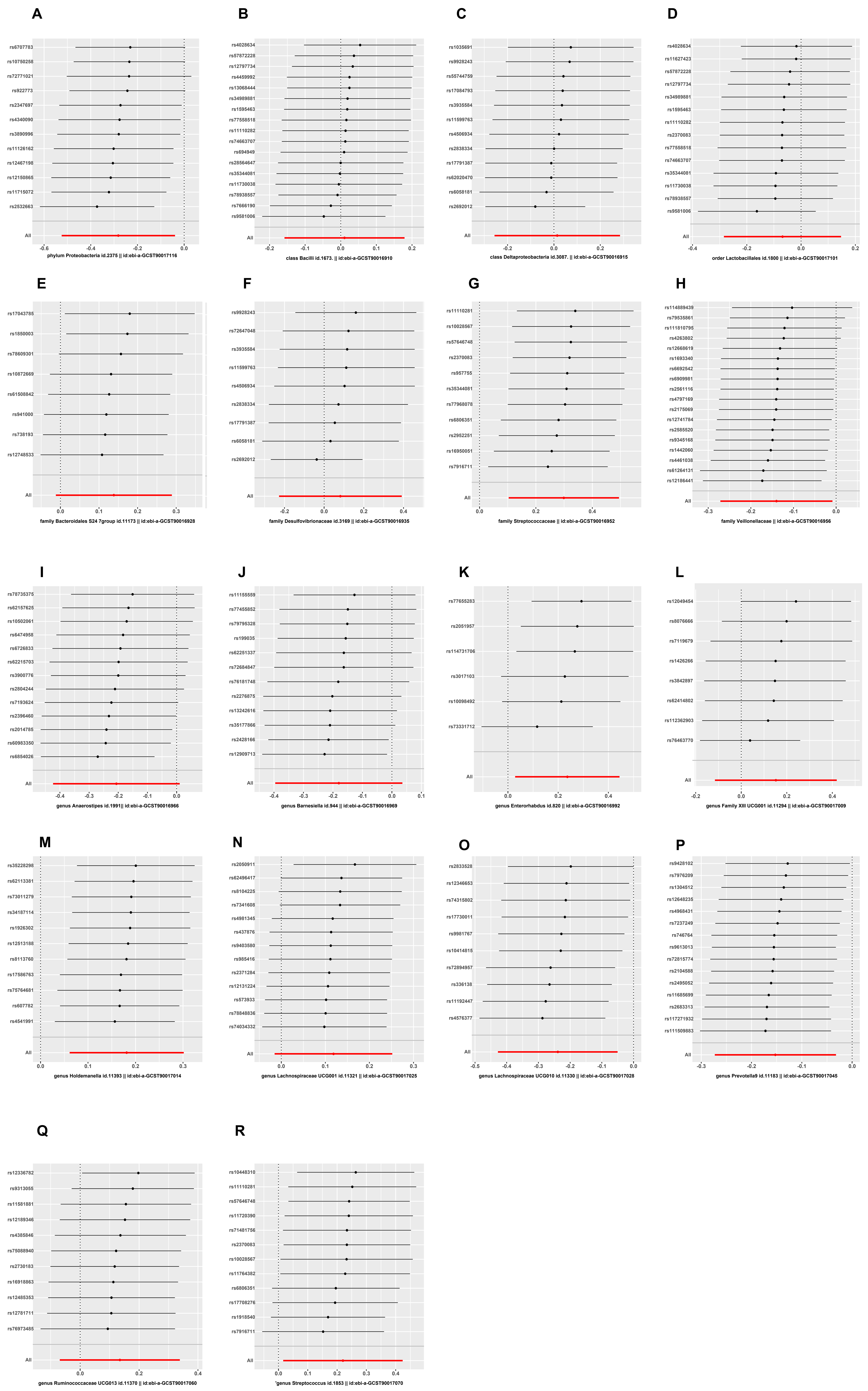

Figure S1 Leave-one-out analysis of gut microbiota on early-onset COPD.

Leave-one-out plot is a useful tool to assess whether the overall effect could be influenced by any specific genetic variant. This method involves sequentially re-evaluating causal estimates after excluding one SNP at a time. The black points in the plot represent the effect estimates after omitting a particular SNP, and the black lines indicate the corresponding 95% confidence intervals. On the other hand, the red points in the plot denote the overall causal effect estimate using a set of SNPs, and the red lines represent the corresponding 95% confidence intervals.

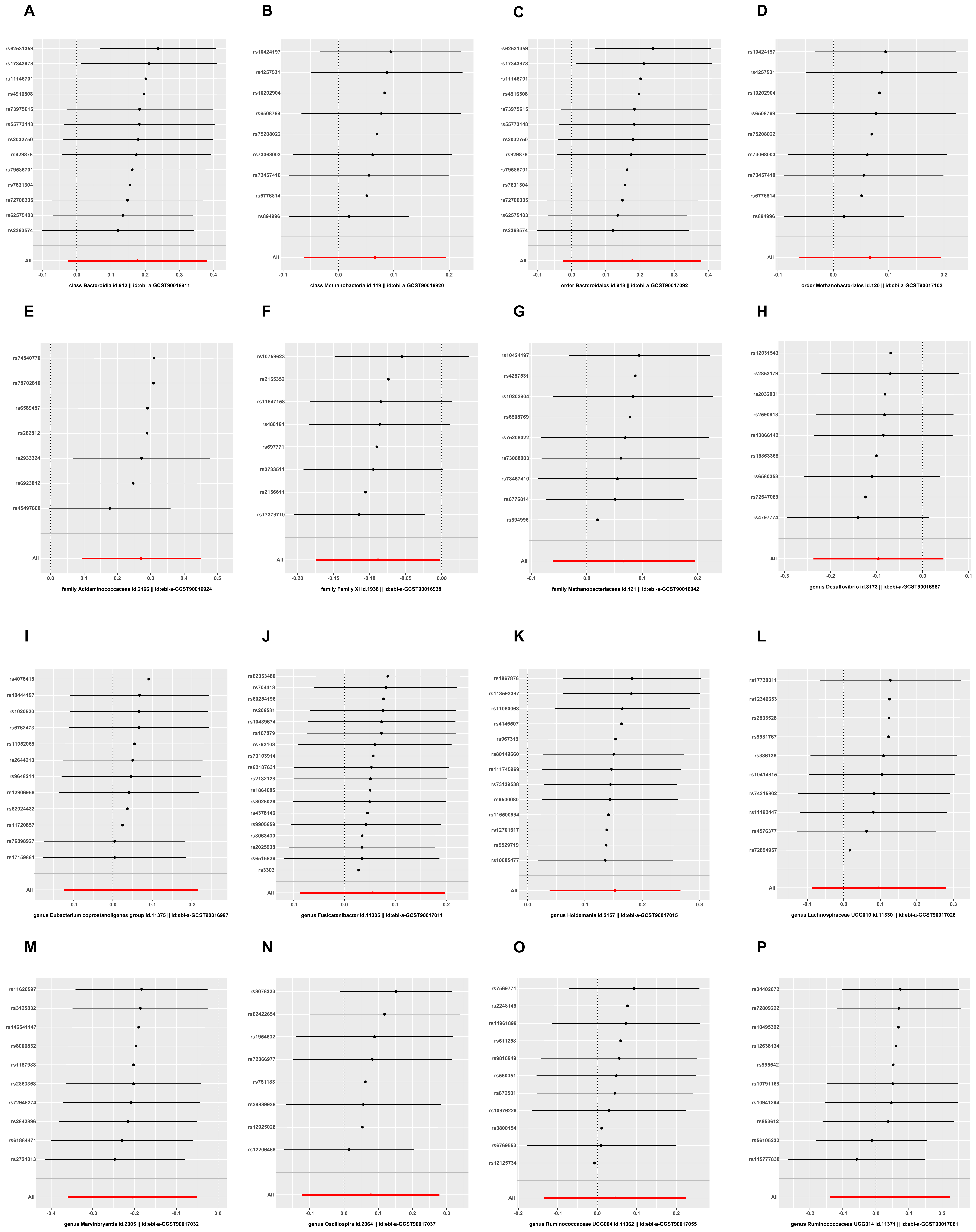

Figure S2 Leave-one-out analysis of gut microbiota on later-onset COPD.

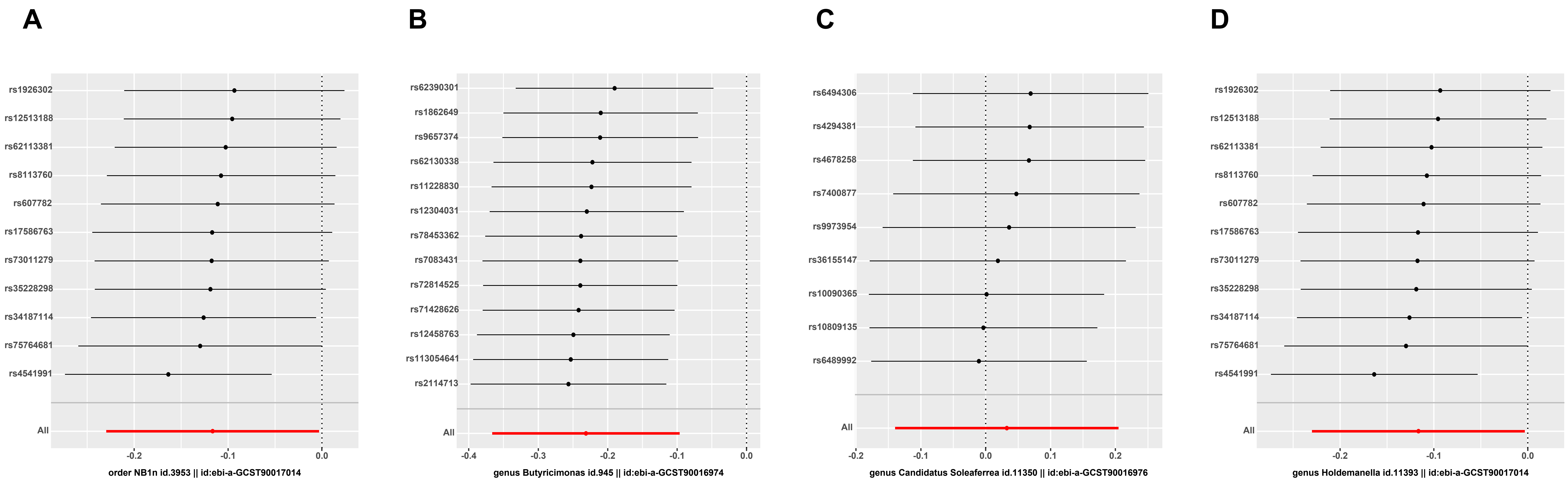

Figure S3 Leave-one-out analysis of gut microbiota on allergic asthma.

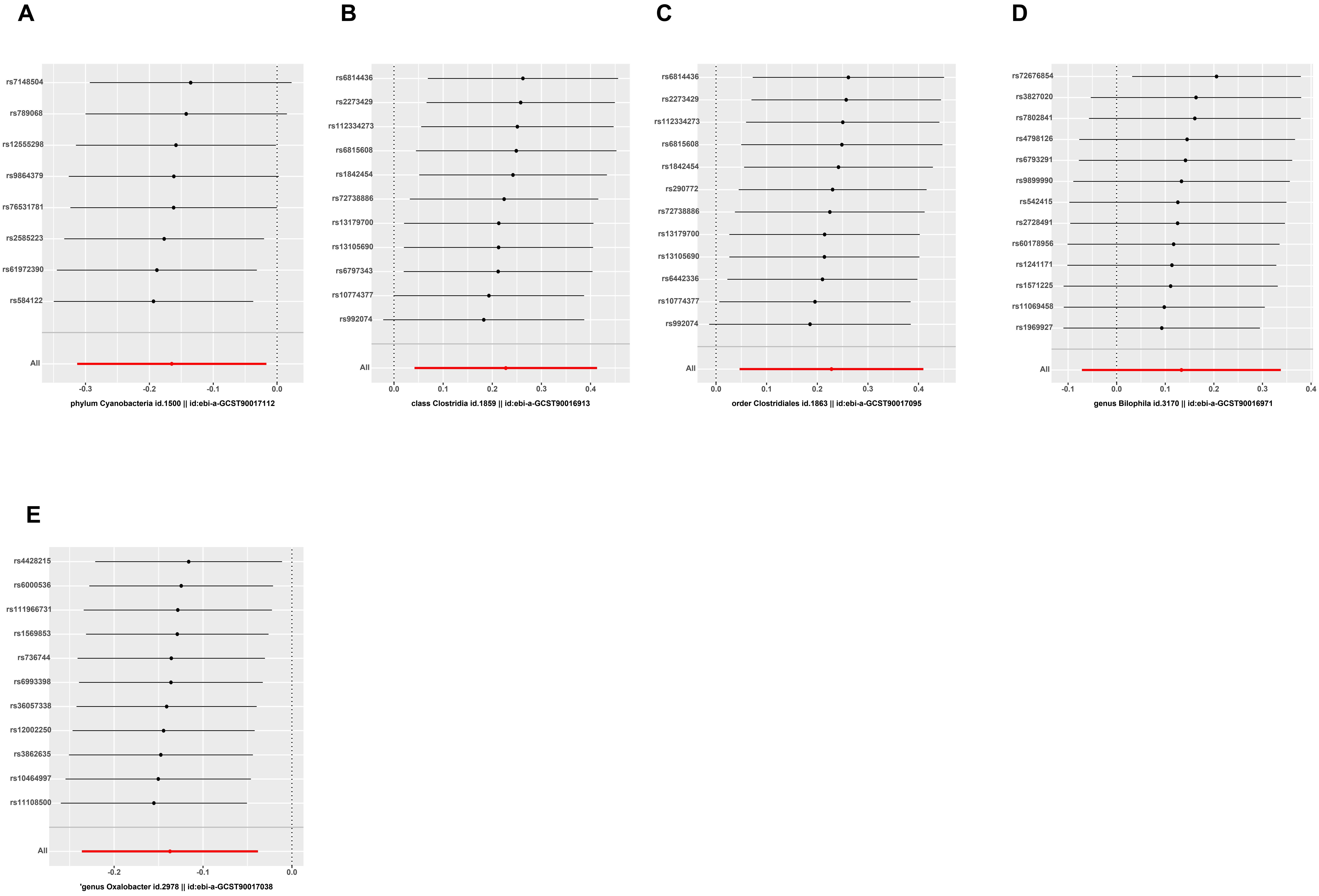

Figure S4 Leave-one-out analysis of gut microbiota on non-allergic asthma.

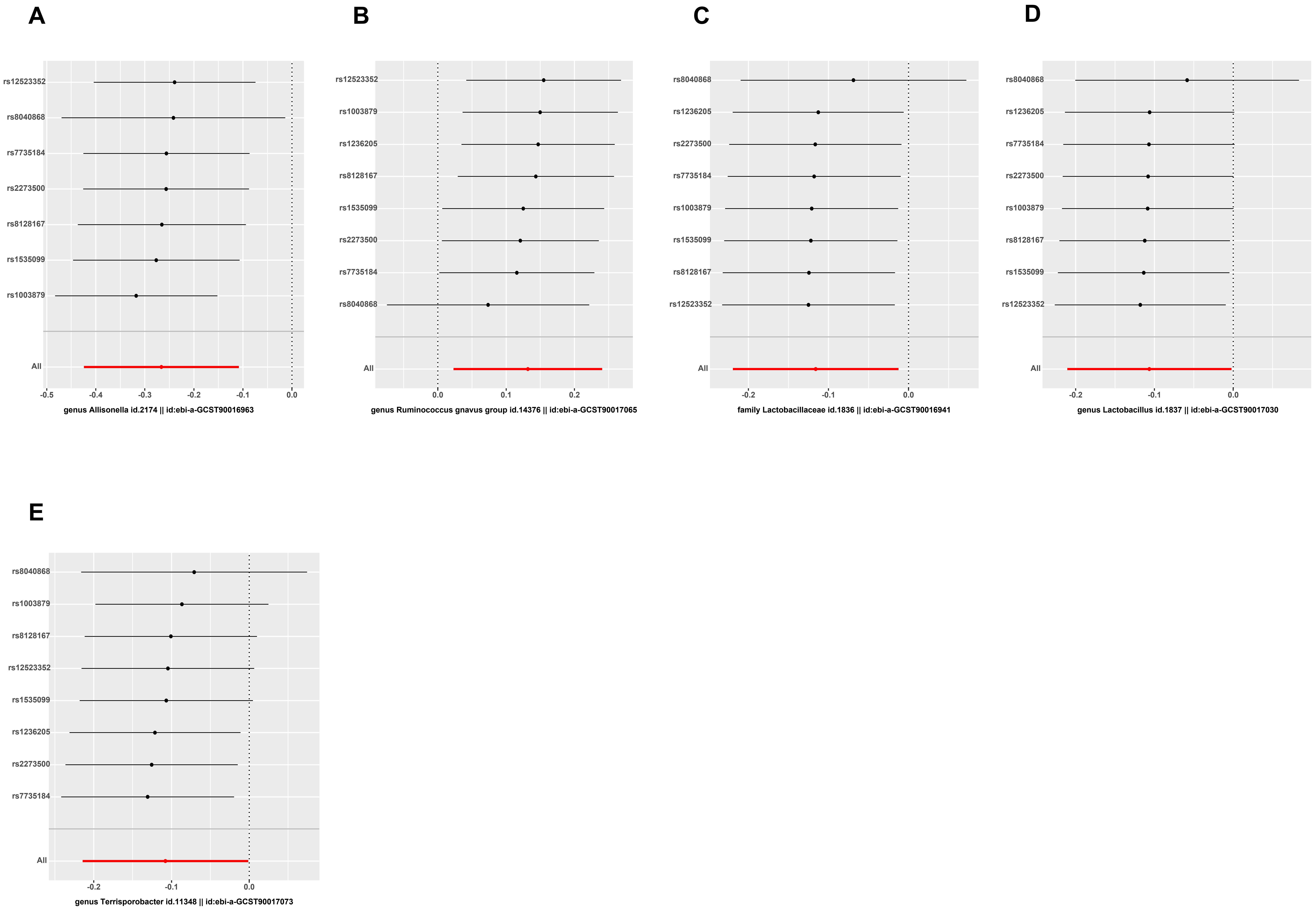

Figure S5 Leave-one-out analysis of early-onset COPD on gut microbiota.

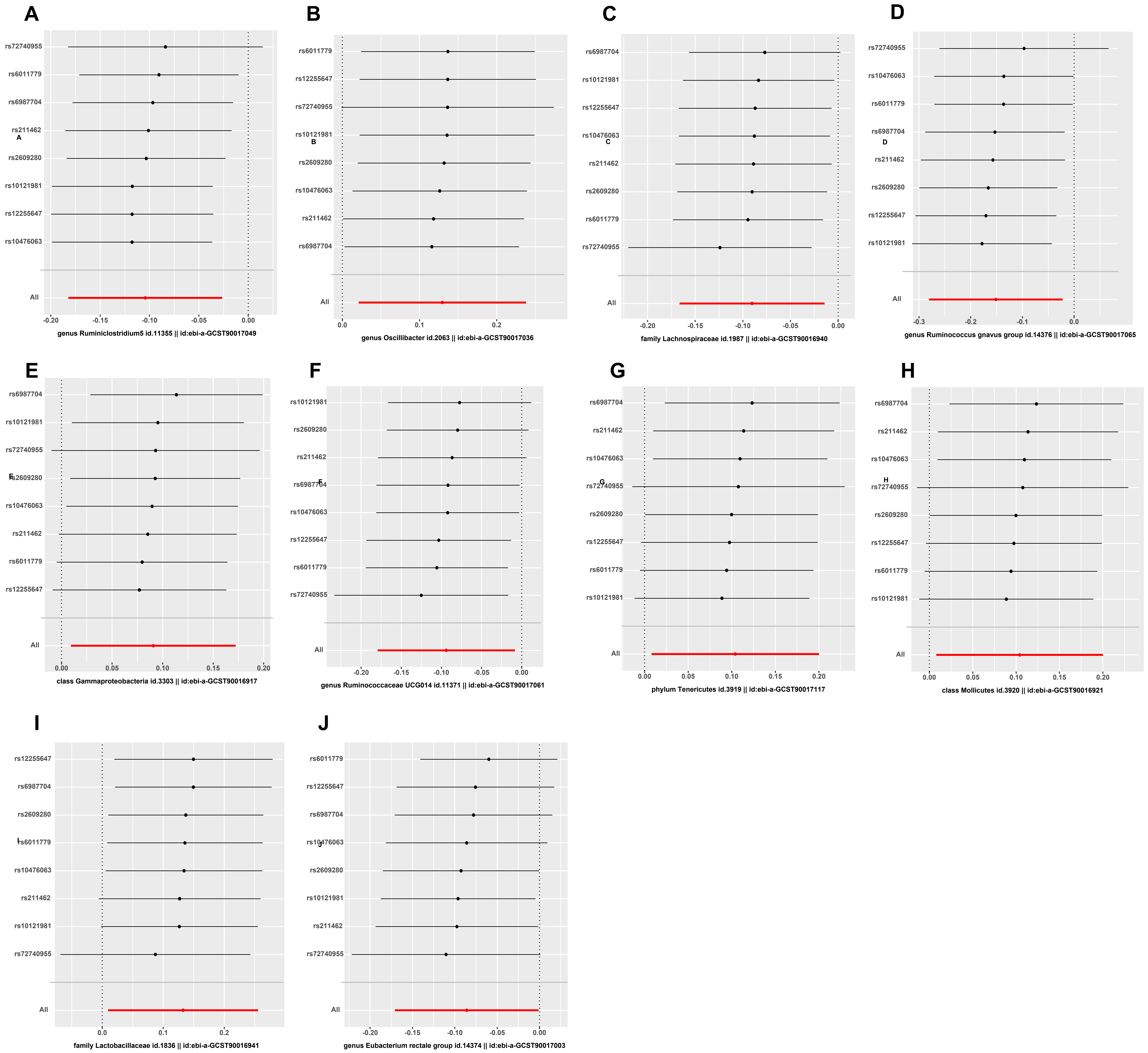

Figure S6 Leave-one-out analysis of later-onset COPD on gut microbiota.

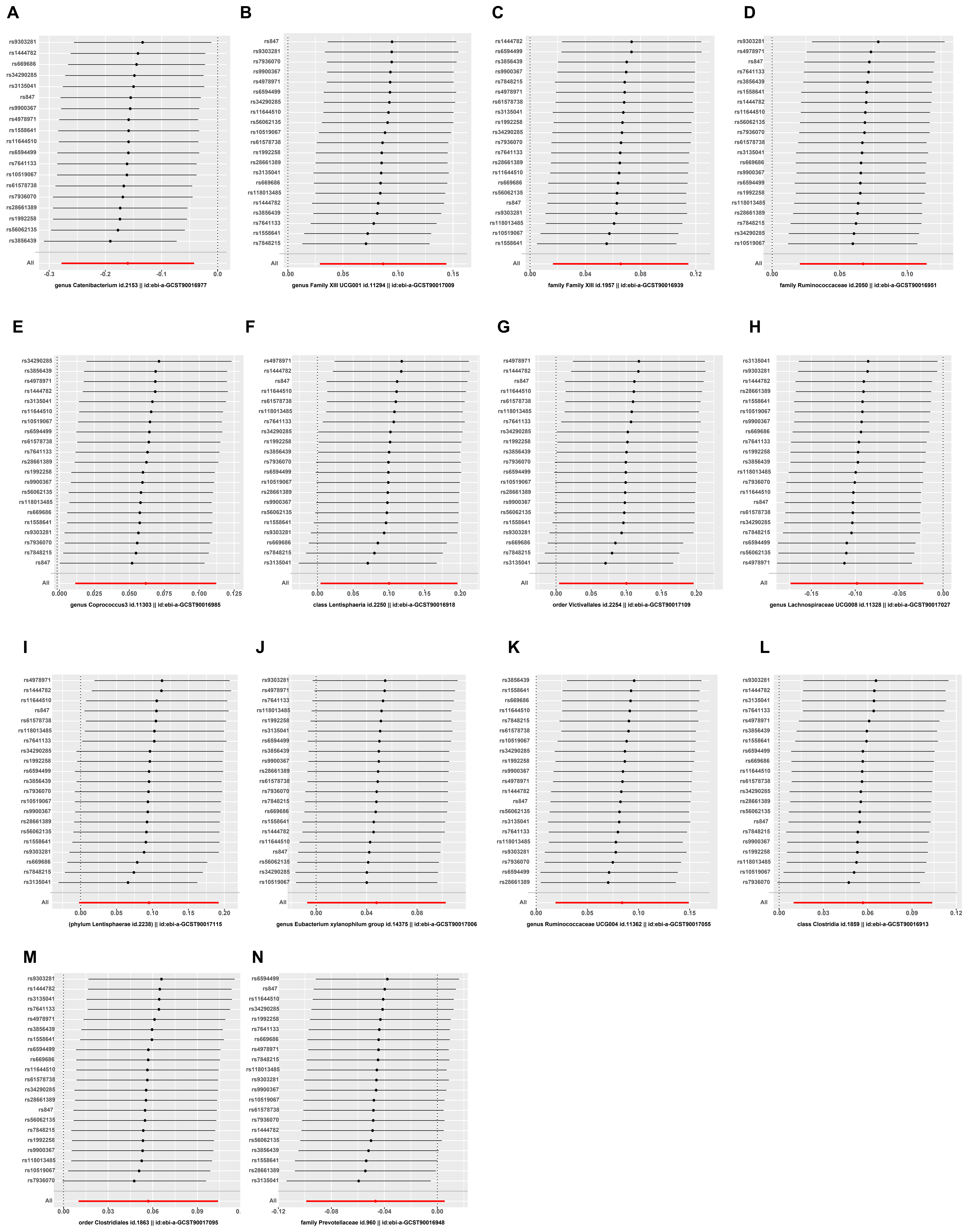

Figure S7 Leave-one-out analysis of allergic asthma on gut microbiota.

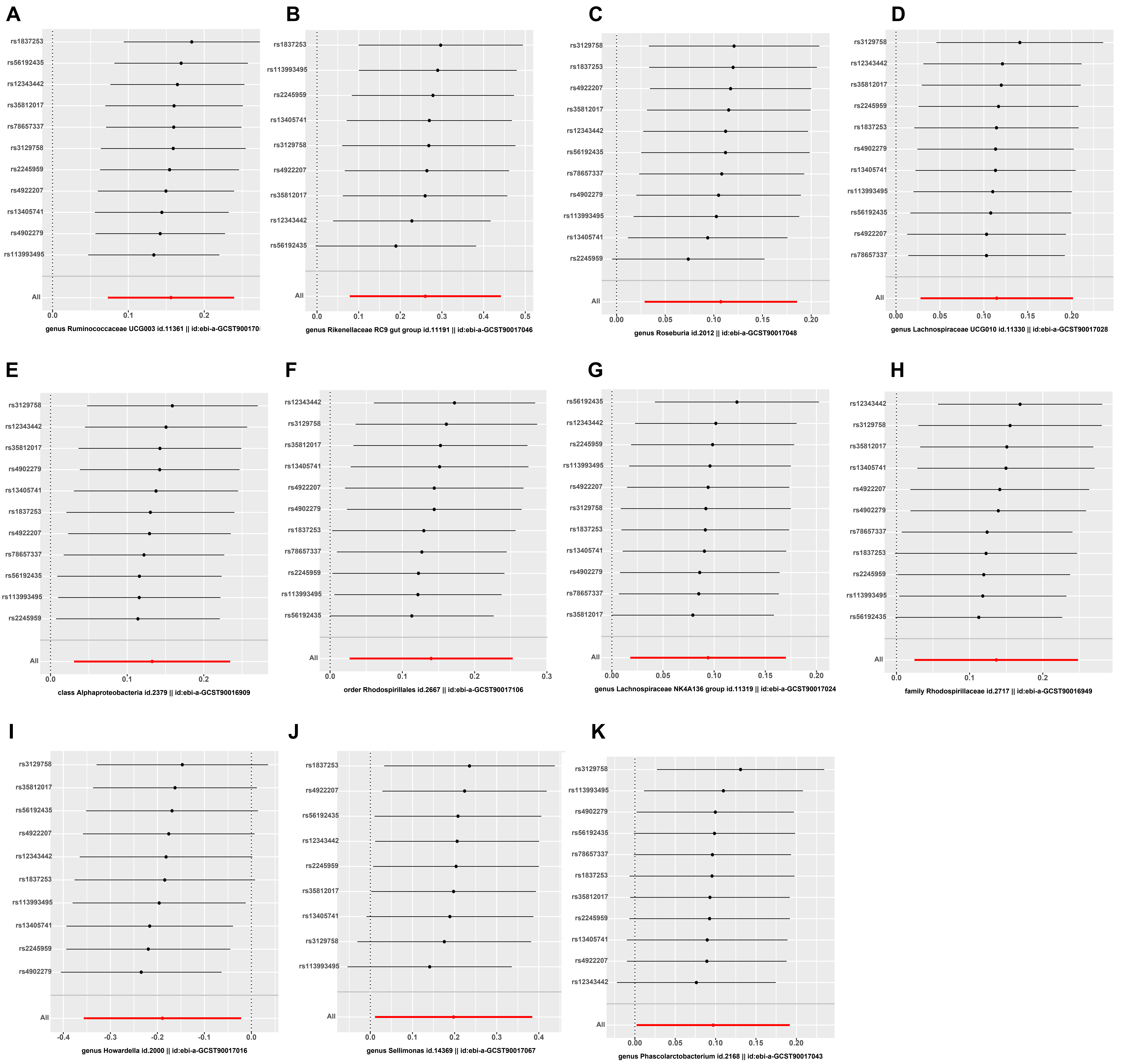

Figure S8 Leave-one-out analysis of non-allergic asthma on gut microbiota.
